# Efficacy of different medications in the treatment of gynaecological tumours: a clinical meta-analysis

**DOI:** 10.3389/fonc.2024.1428937

**Published:** 2024-09-09

**Authors:** Qiaoying Chen

**Affiliations:** Department of Gynecology, Women’s and Children’s Hospital of Ningbo University, Ningbo, Zhejiang, China

**Keywords:** gynaecologic neoplasms, drug therapy, survival rate, non-steroidal anti-inflammatory agents (NSAIDs), metformin

## Abstract

**Background:**

A gynaecological tumour is one of the world’s leading causes of death for women globally. Among women, cancer is the 8th most common cause of death. Since there are no such programmes, the majority of women who are diagnosed with the condition are either in advanced stages or do not respond well to current treatments. Even if patients react to the treatments, they still risk having the cancer return, at which point any further medical intervention is met with resistance.

**Method:**

For this study, we selected the systemic reviews and articles that have the use of different medications used for the treatment of gynaecological tumours.

**Results:**

Regarding metformin use, this study found a positive relationship between higher survival and metformin use. Five of the studies that examined the use of statins revealed a link between statin use and higher overall and/or progression-free survival rates. Individuals on lipophilic and hydrophilic statins would do better. Research evaluating beta-blocker use during neoadjuvant treatment revealed a time-varying effect, with improved survival seen across all users early in the follow-up period. However, only non-selective beta-blocker users demonstrated a correlation with higher survival after five years. One study found that the benefits of aspirin use were significant, but the advantage for continuous users (both before and after diagnosis) was minimal.

**Conclusion:**

Conclusions on the association between gynaecological tumour survival and NA-NSAIDs, metformin, beta-blockers, and aspirin cannot be drawn due to insufficient evidence. However, the vast majority of statin studies have demonstrated that users had higher rates of survival. Bias, however, bias may affect the results of the studies.

## Introduction

One of the leading causes of death among females worldwide is the tumour of the gynaecological system (1). Cancer-related deaths among females are the eighth leading cause. At present, no such population-based programs are screening that help in detecting these cancers early (2). Because there are no such programs, most of the women when diagnosed with the disease in advanced stages or their response towards the treatments that exist is not well. Even if they respond to the treatments, they also face a recurrence of that cancer, which then resists any treatment (3).

Screening for gynaecological cancers or any other kind of cancer is very important. A public health service for the population that seems to be in good health is screening. A test is provided to identify those who are at risk so that more research or therapy can reduce the likelihood of a certain disease or its consequences. Although early discovery of a dangerous ailment might save lives or improve quality of life, screening is not a foolproof procedure and does not ensure protection. The idea behind cancer screening initiatives is that early detection of the disease would lead to better results. Effective illness treatment and a good screening test that is acceptable to the community being tested are prerequisites. There should be few false positives and false negatives overall, and the program should be cost-effective. Different gynaecological cancers include the following:

### Cervical cancer

**Figure 1:**
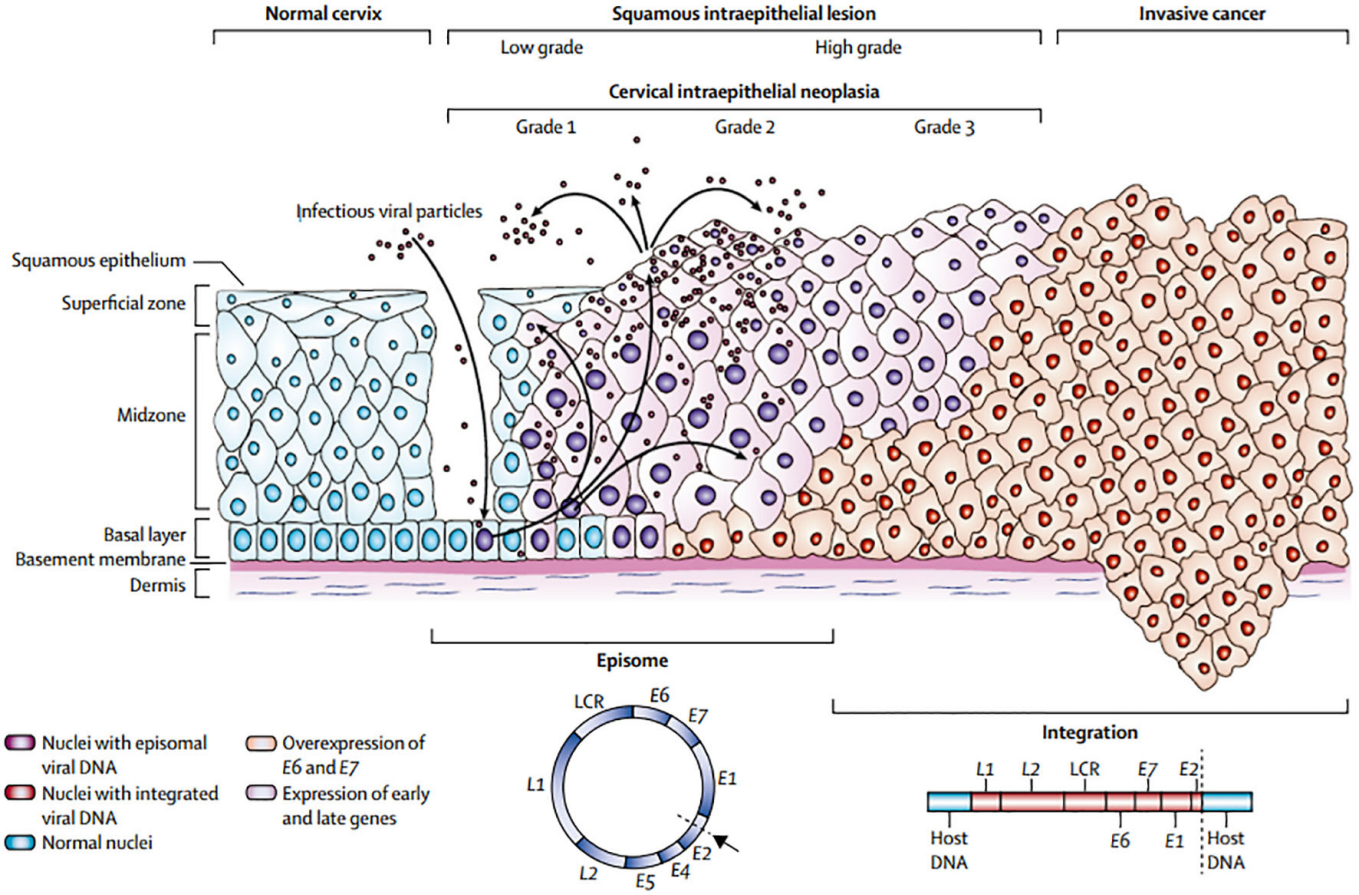
Cervical cancer’s pathogenesis (34).

Cervical cancer is the second most common malignant tumour in women worldwide. Countries with a well-established screening strategy have seen a decrease in the incidence and death of cervical cancer. Cervical cancer pathogenesis is shown in **Figure 1**. 4 The primary method of screening for cervical cancer is exfoliative cytology. By detecting pre-invasive cervical cancer, Papanicolaou (Pap) smear screening significantly lowers the incidence of invasive disease (4). Despite being a useful screening tool, the test’s low sensitivity means that cervical cancer cannot be completely cured. More advanced techniques have been created recently to enhance detection (5).

Cervical cancer is commonly treated with a combination of surgery, chemotherapy, and radiation therapy. Chemotherapy drugs such as cisplatin, paclitaxel, and topotecan are often used either alone or in combination to target and kill cancer cells. Targeted therapies such as bevacizumab have also shown promise in treating advanced cervical cancer by blocking the blood supply to the tumour Immunotherapy drugs like pembrolizumab are being increasingly investigated for their potential in treating recurrent or metastatic cervical cancer by boosting the body’s immune response against the cancer cells. Overall, a multimodal approach combining different treatment modalities has shown the most effective outcomes in managing cervical cancer.

### Fallopian tube and ovarian cancer

In affluent nations, ovarian cancer is the most prevalent cause of cancer-related fatalities among women and the primary cause of death from gynaecological cancer (6). Different histological subtypes of ovarian cancer can be distinguished based on distinct risk factors, cell origin, molecular makeup, clinical characteristics, and treatment approaches (**Table 1**). The need for investigating screening for this disease is supported by strong data that an early diagnosis leads to over 90% 5-year survival rates (7). The screening process is restricted to identifying low-volume illness since no premalignant lesions have been found yet (8).

**Table 1:** Ovarian cancer features based on histology, genetics, and active therapy 8.

Histological subtype	Clinical findings	Genetic characteristics	Treatment options
High-grade serous carcinoma and high-grade endometrioid carcinoma	Can present with peritoneal carcinomatosis, ascites and/or pelvic mass	Deficiencies in homologous recombination (50% of tumours)	Platinum-based chemotherapy and poly(ADP-ribose) polymerase inhibitors
	typically advanced stage at presentation	Associated with *BKCA* and *TP53* mutations	Tumours are initially sensitive to platinum-based chemotherapy. but most patients with advanced-stage cancer will recur
Low-grade serous carcinoma	Presents in younger patients (median reported age: 43-55 years)	Associated with *KRAF* and *BRAF* mutations	MEK inhibitors (currently being tested in clinical traits) and hormonal therapies
	Can be early or late stage at presentation	Tumours have genomic stability	
Low-grade endometrioid carcinoma	Can be associated with endometriosis	Associated with *PTEN*, *ARJDIA* and *PIK3CA* mutations	Possible hormonal therapies (not yet established)
		Can have microsatellite instability	
Clear-cell carcinoma	Can present with parenchymal metastases (in the liver and the lungs)	Associated with *AKID1A* and *PIK3CA* mutations	Immunotherapy agents.
	Can be associated with hypercoagulability and hypercalcaemia		Can be resistant to platinum-based chemotherapy
Mucinous carcinoma	Presents in younger patients and is typically early stage of presentation	Associated with *KRAS* mutations.	Tends to be insensitive to chemotherapy but is still treated initially with cytotoxic chemotherapy

### Endometrial cancer

Ten per cent of all malignancies diagnosed in women are endometrial cancers, which are the most prevalent tumours of the vaginal tract. It is presently not advised to screen for this disorder in the general population or in women who are at elevated risk because of obesity, infertility, diabetes, or tamoxifen usage because the majority of women present with irregular bleeding in an early stage (9). The use of pipeline biopsy for endometrial screening in patients with breast cancer using tamoxifen has been investigated. But before endorsing routine office endometrial biopsies as a common screening procedure for individuals with breast cancer using tamoxifen, further research is needed (10). At the moment, screening is only advised for females who have a genetic susceptibility to the illness as a result of having hereditary nonpolyposis colorectal cancer (HNPCC) syndrome. For these women, their lifelong risk of cancer of the endometrium might range from 40 to 60%. If a preventive colectomy is planned, these women should be advised to undergo a prophylactic hysterectomy and a bilateral salpingo-oophorectomy, particularly if they have previously had children. According to certain research, women who have or are at risk of developing HNPCC do not substantially differ in their chances of surviving endometrial cancer from those in the general population (11, 12).

### Vulval cancer or vaginal cancer

There isn’t much information in the literature on these uncommon malignancies. Elevations of the urine core fragment of the HCG beta subunit, SCC, and tissue polypeptide specific antigen (TPS) have been seen in certain investigations. No research has been done on the benefits of screening (12).

When the diagnosis of these conditions is made in females, less than 50% of the females survive for at least five years. Many studies have been published attributing how the use of common medications impacts the survival of these patients (3). Commonly used medications used by these patients include metformin, beta-blockers, statins, aspirin as well as non-steroidal anti-inflammatory medicines (NSAIDs) (13). Many systemic reviews have been published that demonstrate these medicines positively affect the survival of patients with tumours and improve survival chances. Also, there are a few studies which also suggest that the effects that these medicines bring can also vary because of the subtypes of the medication like statins of lipophilic or hydrophilic nature, or selective beta blockers or non-selective beta blockers (14). The period to which these medicines are being used also affects the impact that they brings (15).

More than two-thirds of patients with advanced cancer report having significant pain, and as many as half of them say their pain is not well managed. Patients with gynaecologic oncology may also feel acute discomfort due to the burden of their disease or the course of their cancer therapy (16).

Regardless of speciality, gynaecologic oncologists may help stop the opioid crisis by treating patients’ pain with awareness and purpose. This can start with developing improved recovery protocols and performing a greater percentage of minimally invasive operations to handle gynaecologic cancers surgically (17).

Gynaecologic oncologists should provide a pain evaluation to individuals with either acute or ongoing cancer or pain linked to therapy at every visit. The location of the pain, aggravating and mitigating variables, current therapies, and any prior treatments should all be covered in this evaluation. Clinicians should also check if drug demands are rising, steady or decreasing. It is important to rule out recurring or progressive illness in individuals presenting with new or worsening pain (18).

In addition, the administration of beta-adrenergic receptor antagonists, or beta-blockers, during cancer treatment has been suggested to have potential benefits based on experimental and epidemiological findings. This may be because the sympathomimetic neurotransmitters norepinephrine (NE) and epinephrine (E) are inhibited in their ability to act. These neurotransmitters may play a significant role in the development of secondary tumours and may be involved in some of the eight recognized characteristics of cancer, such as metastasis (19).

Since beta blockers are thought to be inexpensive, safe, and effective medications, it would be extremely advantageous to explore any possible adverse effects before using them (19).Nevertheless, data from relevant epidemiological studies have yielded conflicting results, and it has been proposed that immortal time bias—a period of cohort follow-up during which a population cannot experience an event because of the definition of drug exposure—may be partially to blame for the apparent discrepancies in study results (20).

Adults in the US take statins often to decrease their low-density lipoprotein (LDL) levels of cholesterol and avoid cardiovascular disease; in 2012, 28% of those over 40 reported using a statin. There have been documented non-cardiovascular advantages of statin usage, such as possible anti-tumour actions in a broad range of cancers. Statin users had a substantial 15% lower incidence of mortality from cancer and a 15% lower frequency of death from any cause, according to population-based observational research of 295,925 individuals in the Danish Cancer Registry (21).

We found a substantial 30- 40% increase in overall survival when statin usage was independently associated with this large prospective cohort of older women with ovarian cancer. This is consistent with smaller retrospective datasets that were previously reported and showed increased overall and disease-free survival in patients receiving concomitant statin medication for primary peritoneal, fallopian tube, or epithelial ovarian carcinomas (19).

The survival rate specific to ovarian cancer was statistically substantially higher for women who reported recent use of aspirin and non-aspirin nonsteroidal anti-inflammatory drugs (NSAIDs) in the time following diagnosis for the NHS recent for the NHSII, with identical timeframes for the evaluation of NSAIDs. These findings call for more research, and if they are validated, it could be interesting to evaluate the use of anti-inflammatory drugs after diagnosis in randomized trials in addition to conventional ovarian cancer treatments to enhance patient outcomes (22).

Another study suggested that Even though there is significant variability, cancer patients who take metformin have longer survival than those who do not (23). Met24 Metformin is linked to non-significantly higher survival times for malignancies of the prostate, lung, liver, larynx, and bladder, but substantially longer survival times for breast, colorectal, endometrial, and ovarian cancers (24). The main drawback is the high degree of study heterogeneity and the paucity of data for some cancer types (25).

## Methods

For this study, we selected the systemic reviews and articles that have the use of different medications used for the treatment of gynaecological tumours. Controlling for bias and compensating for missing values were explored in our study, which in turn led to a more statistically rigorous article and a more conclusive article.

### Search strategy

The search was done on Embase (Elsevier), PubMed (National Library of Medicine, Web of Science and Google Scholar. Some research was also done on the list of references of the articles that were eligible for this study. Each article was thoroughly studied for relevant information and then the information was extracted from it and stored in a secure database.

### Eligibility criteria

#### Exposures

Multiple terminologies were used throughout the studies regarding the use of medications in tumours. However, we classify the exposures as ever or never use of any sort of medications before or after the diagnosis.

#### Study population

The population of this study were the women who have experienced any gynaecological tumour (ovarian, fallopian tube or primary peritoneal) and went through the use of medicine for it.

### Study selection and data extraction

All the studies that were identified were stored in an endnote file. Each study was assessed for its eligibility and after reviewing the titles and the abstracts of different studies. For the studies that remained, we obtained full-text papers wherever possible, and we eliminated those that had ineligible individuals or samples. When a research or dataset had numerous publications, we included the report with the biggest sample size, the most comprehensive data, or the most extended follow-up period. For this purpose, we utilized 10 studies to be included in this analysis and they were studied for their characteristics and hazard ratios (95% CI). We tried our best to resolve any type of discrepancies from the studies. The disagreements of reviewers were put into question throughout the study duration, and conflicts of interest were addressed during the process of data extraction so that no questions could be raised after the study was completed.

### Quality assessment

A reviewer independently evaluated each study’s quality using the Cochrane ROBINS-I method, which assigns a risk of bias (ROB) score of low (quality comparable to a randomized clinical trial), moderate, serious, or critical. Since none of the included research was deemed to have low ROB, we categorized the investigations based on whether or not they had substantial ROB. The Cochrane ROB, as a revised version of the current evidence appraisal, categorizes the quality of evidence in the included literature more accurately, increasing the overall quality of this paper.

### Statistical analysis

To create pooled hazard ratios (pHR), we employed random-effects models and the inverse variance approach. When required, we estimated the pertinent confidence bounds using the provided P-value (P). To avoid all kinds of reverse causation from the studies, in those studies where hazard ratios were available for both times diagnosis and post-diagnosis, we utilized the data of pre-diagnosis assuming that the patient must have continued the use of medication after the diagnosis as well. The analysis was done using SPSS version 22 and STATA 15.

## Results

### Metformin

**Figure 2:**
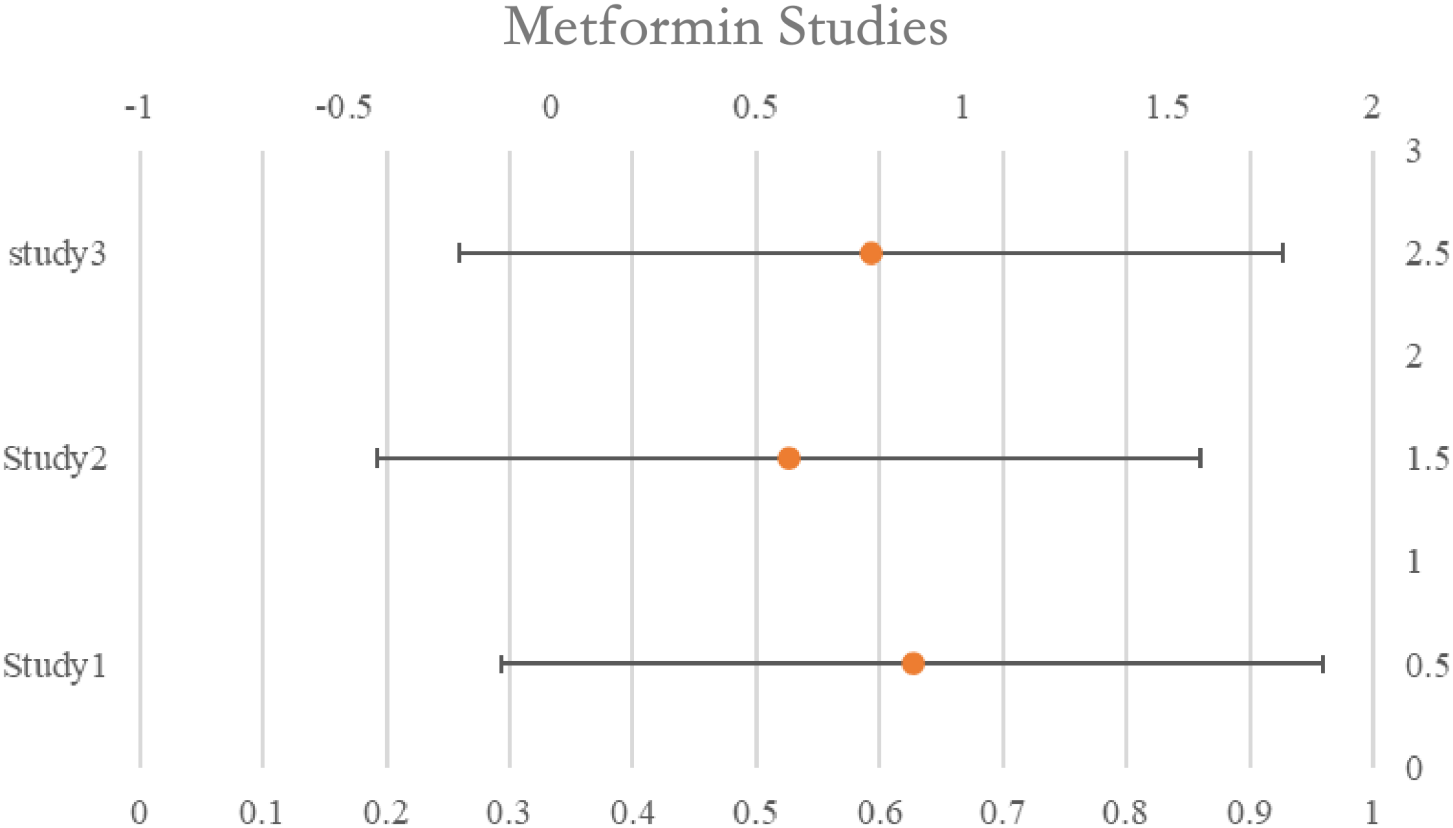
Rest plot for the meta-analysis regarding hazard ratio among the patients using metformin.

The survival rates of women taking metformin were compared to those of non-users who were either women with diabetes, women without diabetes, or women with both diabetes and non-diabetes combined. In all, three studies found a correlation between the usage of metformin and increased survival; nevertheless, they were all thought to have ITB. The two trials that were graded as ITB-free indicated that there was no overall survival advantage linked to the use of metformin; however, the research that assessed usage within six months before or following diagnosis found that metformin users had improved survival 30 months after diagnosis. The combined estimate of all research, including those classified as possibly having ITB, indicated a positive correlation between metformin usage and better survival (pHR: 0.66, 95%CI: 0.44–1.00) as well as better survival among users (pHR: 0.45, 95%CI: 0.33–0.60). See **Table 2** for details and **Figure 2**.

**Table 2:** Metformin studies and its characteristics.

Metformin Studies	Population	HR (95% CI)
USA 2017 (Garcia)	2291	0.88 (0.66, 1.17)
USA 2012 (Romero)	341	0.58 (0.23, 0.83)
Israel 2016 (Bar)	2016	0.78 (0.40, 1.42)

### Statin

**Figure 3:**
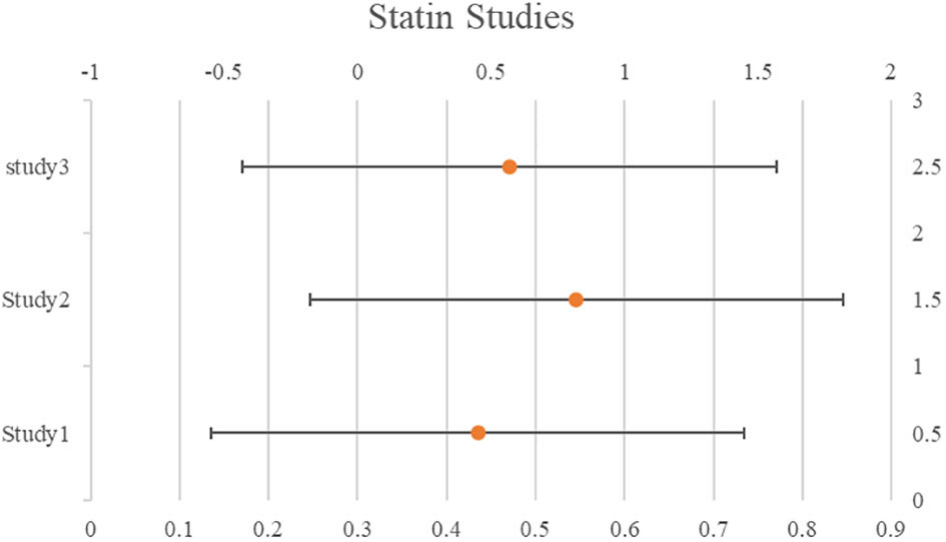
Forest plot for the meta-analysis regarding hazard ratio among the patients using Statin.

Among the studies that were focused on the use of statins, 5 studies suggested a correlation between statins and increased survival rates overall and/or progression-free. A correlation between statins and increased survival was revealed by pooling the data from the eight ITB-free trials (pHR: 0.76, 95%CI: 0.68–0.85). Publication bias was not evident (P=0.059). Based on the exposure timing, we categorized the research. A relationship between statin usage and increased survival was revealed by the calculated pHRs based on pre-diagnosis use (three studies, pHR: 0.77, 95%CI: 0.67–0.87), perioperative use (two studies, pHR: 0.60, 95%CI: 0.48–0.72), and post-diagnosis use (three studies, pHR: 0.81, 95%CI: 0.74–0.89). The studies also revealed a correlation between statin use and a lower death rate (pHR: 0.63, 95%CI: 0.37–1.09). Two of the three studies that examined the survival results of lipophilic and hydrophilic statins independently were assessed as ITB-free; yet, one of the studies categorized atorvastatin as hydrophilic even though its characteristics are mostly lipophilic. The results of the three trials indicated that patients using hydrophilic and lipophilic statins would fare better. See **Table 3** for details and **Figure 3**.

**Table 3:** Statin studies and its characteristics.

Statin Studies	Population	HR (CI 95%)
USA 2008 (Elmore)	126	0.45 (0.23, 0.88)
Belgium 2017 (Couttenier)	4895	0.82 (0.72, 0.93)
China 2016 (Chen)	60	0.57 (0.21, 1.55)

### Beta-blockers

**Figure 4:**
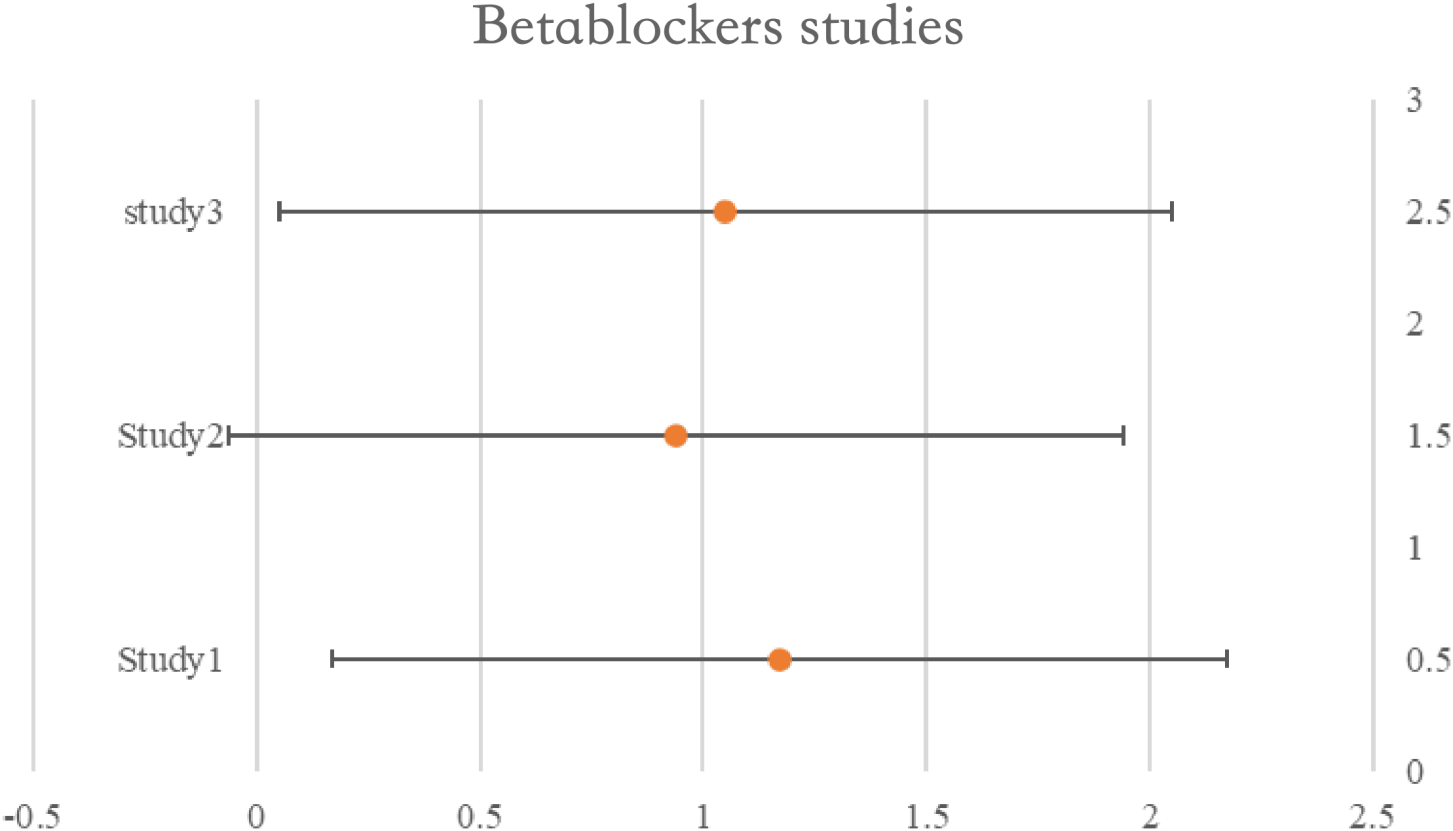
Forest plot for the meta-analysis regarding hazard ratio among the patients using beta-blockers.

A time-varying impact for beta-blockers was shown in research assessing usage during neoadjuvant treatment, with better survival observed amongst all users early in the follow-up period. However after five years, only non-selective beta-blocker users showed a relationship with increased survival, and the association was larger in women without hypertension. Another study had provided HRs for exposures both before and after diagnosis. A meta-analysis of six ITB-free trials that included the pre-diagnosis estimate produced a pooled HR of 1.07 (95%CI: 0.96–1.21), indicating no survival advantage for beta-blockers. There was no correlation between usage and survival, according to the combined findings of the three trials that assessed PFS (pHR: 0.97, 95%CI: 0.78–1.20). Nevertheless, combining the findings from the other two trials assessing perioperative usage revealed a potential link between use and enhanced PFS (pHR: 0.87, 95%CI: 0.69–1.09) and overall (pHR: 0.82, 95%CI: 0.60–1.12). See **Table 4** for details and **Figure 4**.

**Table 4:** Beta-blocker studies and their characteristics.

Beta-blockers studies	Population	HR (CI 95%)
Denmark 2013 (Johannesdottir)	6626	1.17 (1.02, 1.34)
Germany 2017 (Heltz)	801	0.94 (0.69. 1.29)
South Korea 2018 (Baek)	866	1.05 (0.80, 1.37)

### Aspirin & NSAID’s

One of the studies assessed survival rates using data from both pre- and post-diagnosis periods. When the HRs assessing pre-diagnosis usage were included in the initial meta-analysis, we saw no correlation between aspirin use. Use both before and after diagnosis was evaluated in two trials.

**Figure 5:**
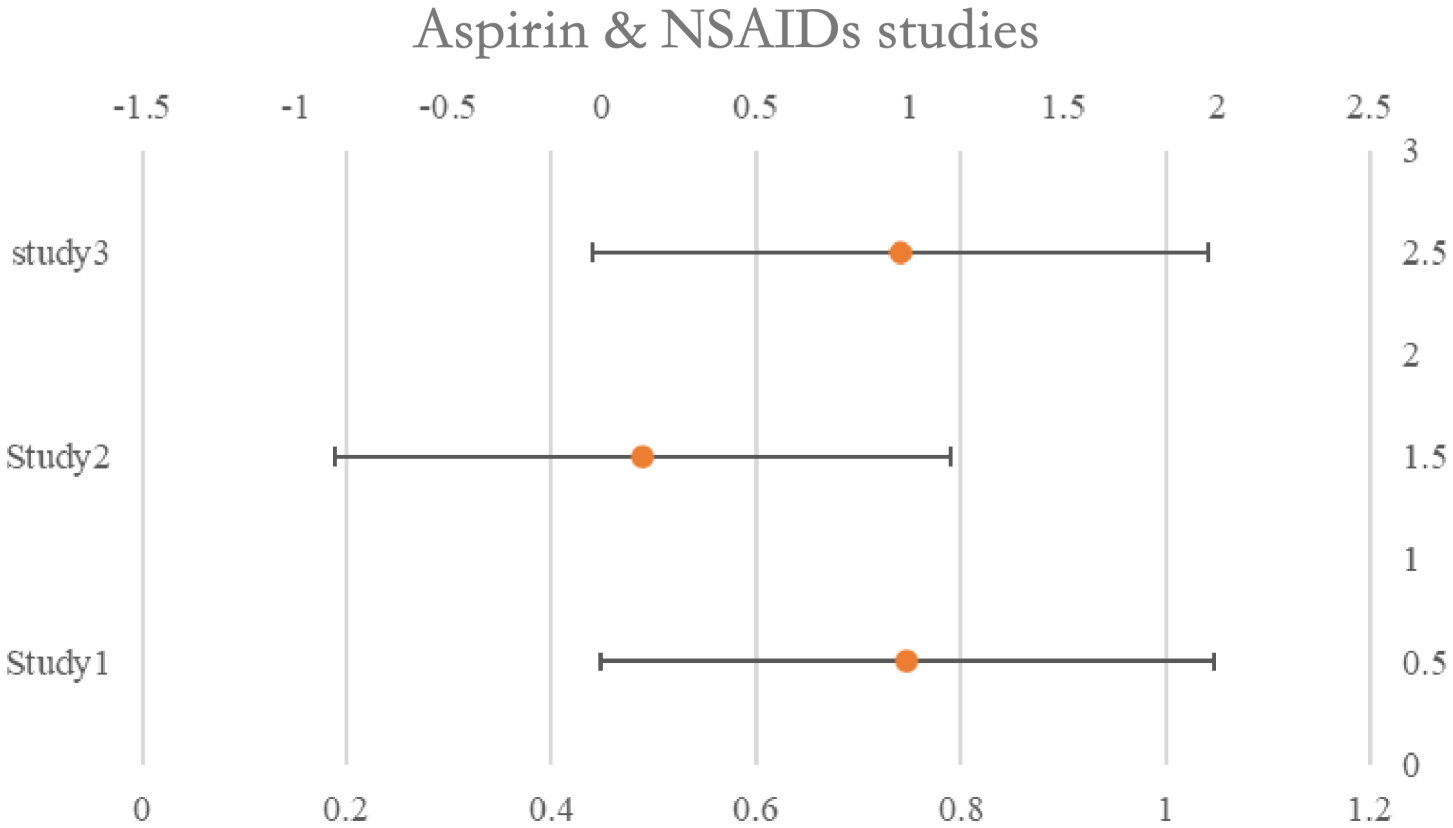
Forest plot for the meta-analysis regarding hazard ratio among the patients using Aspirin & NSAIDs.

Aspirin, HR: 0.44, 95%CI: 0.26–0.74; NSAIDs, 0.46, 95%CI: 0.29–0.73) and post-diagnosis survival benefit was shown to be significant in one study, while the advantage to continuous users (pre- and post-diagnosis) was low. The second research found no evidence that ongoing low-dose aspirin treatment improved overall survival (HR: 1.01, 95%CI: 0.84–1.22). See **Table 5** for details and **Figure 5**.

**Table 5:** Aspirin & NSAIDs studies and their characteristics.

Aspirin & NSAIDs studies	Population	HR (CI 95%)
USA 2018 (Merritt)	1022	0.99 (0.79, 1.25)
USA 2017 (Wield)	77	0.13 (0.02, 0.95)
Denmark 2018 (Verdoot)	4117	0.97 (0.87, 1.08)

## Discussion

Studies on statin usage showed that users might live longer (26). The combined findings of beta-blocker trials indicated a potential advantage linked to perioperative usage but no improvement in overall survival (27). The small number of trials revealed no survival advantages linked to metformin, and the evidence supporting a relationship between aspirin and NSAIDs was weak, although more research is needed. Studies addressing the possibility of Immortal Time Biased were generally more likely to demonstrate links with the drugs and increased survival (28).

An essential enzyme in the mevalonate pathway, 3-hydroxy-3-methylglutaryl-coenzyme A (HMG-CoA) reductase, is inhibited by statins25 The primary byproduct of the mevalonate pathway in liver cells, cholesterol, is reduced by this restriction, but it also has an impact on several non-sterol side products necessary for cell division, survival, and repair. Studies conducted *in vitro* have revealed that certain cancer cells, such as those from the breast and ovary, have abnormally elevated expression of the HMG-CoA reductase gene. Tumour cells can proliferate more rapidly and survive longer than normal cells due to altered metabolism. One proposed mechanism is that statins, by inhibiting the mevalonate pathway and reducing its downstream products, may induce apoptosis, or cell death, thereby impeding tumour growth. Additionally, preclinical studies have demonstrated that combining statins with chemotherapy drugs enhances the effectiveness of cancer treatment (24).

All things considered; these findings imply that it would be worthwhile to look at the possible effects of statins in a study that also assesses whether statins might increase survival rates among women without hyperlipidaemia.

Experimental research has indicated that using beta-blockers during surgery may improve survival, mostly by limiting the possibility of cell migration and metastases (29).

A limitation of the included studies is that they could only assess medication use in women with the specific medical condition necessitating the medication (18).

Furthermore, the severity of the ailment being treated as well as the prognosis of the cancer may have an impact on the usage of chronic illness drugs during cancer therapy or following diagnosis, both of which can also have an impact on survival rates. Because the expected benefit would be small, women with poorer cancer prognoses could decide not to start preventative medicine or stop taking it altogether. (30) This may pose a special challenge for assessments of new usage after diagnosis and help to explain some of the substantial correlations observed between the new use of NSAIDs and aspirin after diagnosis and increased survival (31).

Certain confounding factors also impose a tricky situation for the researcher to conduct the research. Such confounding factors like parity and oral contraceptive use can also play a vital role in the medication used for the treatment of gynaecological cancers.

## Conclusion

In conclusion, there is not enough data available to make any judgements on the relationship between gynaecological tumour survival and metformin, beta-blockers, aspirin, and NA-NSAIDs. On the other hand, the majority of research on statins has shown that users had better survival rates. However, bias might have an impact on observational study outcomes. Moreover, they are only able to evaluate statin usage in women who are prescribed these drugs, often for hypercholesterolemia; they are unable to evaluate statin use in women whose cholesterol levels are normal. Randomized studies are necessary to ascertain if administering statins as an adjuvant therapy to women with gynaecological cancer during or after chemotherapy could enhance their likelihood of survival.

## Data Availability

The original contributions presented in the study are included in the article/supplementary material. Further inquiries can be directed to the corresponding author.
